# The prevalence of stress and burnout in UK emergency ambulance service workers and its impact on their mental health and well-being

**DOI:** 10.29045/14784726.2021.3.5.4.62

**Published:** 2021-03-01

**Authors:** Elisha Miller

**Affiliations:** Yorkshire Ambulance Service NHS Trust

## Abstract

**Aims:**

To assess the prevalence of burnout among emergency ambulance service workers in one ambulance station; to determine if there are any variances based on socio-demographic information such as gender, clinical grade and length of service; to examine the distinctions between personal, work-related and patient-related burnout; to identify current workplace interventions to reduce stress and burnout that will improve mental health and well-being.

**Methods:**

Mixed methods – the Copenhagen Burnout Inventory (CBI) was utilised, measuring burnout across three domains (personal, work-related and patient-related) alongside collecting demographic information such as gender, role, full-time or part-time employment and length of service. A free-text space was available to provide opinions on causes of burnout and on how current practice can be improved; these were analysed via thematic analysis.

**Results:**

Seventy-eight staff members completed the questionnaire. These were: 16 emergency care assistants, 15 technicians and 47 paramedics. Thirty-eight (48.7%) staff members experienced personal burnout, 42 (53.8%) experienced work-related burnout and 29 (37.1%) experienced patient-related burnout. It was found that those most at risk of burnout were full-time male employees with more than 10 years’ experience and employed within a paramedic position. Six themes were identified through thematic analysis: unnecessary callouts, shift patterns, support options, management, sickness absence and job demands.

**Conclusion:**

Findings suggest that burnout is prevalent within the ambulance service environment and can result in long-term sickness absences and declining mental health. This requires further investigation into causation alongside consideration of preventative measures and interventions to improve ambulance service staff well-being while increasing staff resilience to prevent burnout. Support for managers in recognising symptoms of burnout is also imperative because providing them with the training to recognise a mental health issue, interpret it and promptly treat it can mean the difference between sickness absences, future PTSD, unwell staff presenting at work and staff feeling valued and supported by management. The introduction of mandatory counselling and well-being sessions was also recommended by participants to improve staff mental health and well-being and reduce instances of work-related burnout, while providing financial advantages to the ambulance service with a reduction in additional overtime and sickness payments.

